# Experimental Evidence on the Nature of the Antigen in the Direct Agglutination Test for Visceral Leishmaniasis

**DOI:** 10.4269/ajtmh.19-0784

**Published:** 2020-03-02

**Authors:** Vera Kühne, Ruben Verstraete, Xaveer van Ostade, Philippe Büscher

**Affiliations:** 1 Institute of Tropical Medicine, Antwerp, Belgium;; 2 University of Antwerp, Antwerp, Belgium

## Abstract

The direct agglutination test (DAT) for visceral leishmaniasis (VL) is the serodiagnostic test for VL that has the most robust sensitivity and specificity in the field across all endemic regions. It is based on trypsin-treated and formaldehyde-fixed whole promastigote cells from *Leishmania donovani*. The exact identity and nature of the epitopes on the DAT antigen that cause agglutination with VL patients’ sera are currently unknown. In this study, we performed antigen-inhibition studies which revealed that lipophosphoglycan (LPG) and the DAT antigen share epitopes. Antibody inhibition with a monoclonal antibody directed against the phosphoglycan repeat epitope of LPG showed that this is not the epitope that reacts with human sera. Oxidation of carbohydrates by sodium metaperiodate did not alter the reactivity of human sera with the DAT antigen and LPG. This indicates that carbohydrates do not play a role in the reaction of the DAT antigen with antibodies in serum from VL patients, and that they also are not involved in the reaction of LPG with the same serum. We conclude that the noncarbohydrate moiety of LPG, that is, the core–anchor fragment, and potentially other noncarbohydrate epitopes on the surface of the DAT antigen are responsible for its agglutination with antibodies from VL patients. As LPG plays a role in the DAT reaction, this could facilitate the following: 1) incorporation of LPG, preferably the synthetic version of the core–anchor fragment, into an immunochromatographic test format that is more adapted as a point-of-care test (short incubation, little training, and equipment needed) than DAT and 2) enhancing the quality control for the production of the DAT antigen.

## INTRODUCTION

Visceral leishmaniasis (VL) is a fatal disease that is caused mainly by protozoan parasites of the species *Leishmania donovani* and *Leishmania infantum*. The direct agglutination test (DAT) for the detection of VL is a highly specific and sensitive antibody detection test for use in resource-poor settings. Its diagnostic sensitivity and specificity are high across all geographic regions endemic for VL with 94.8% (95% CI: 92.7–96.4%) sensitivity and 97.1% (95% CI: 93.9–98.7%) specificity.^1^ The antigen in the DAT consists of whole promastigote cells from a Sudanese *L. donovani* isolate (MHOM/--/SD/1-S) that have been trypsin treated, formaldehyde fixed and stained with Coomassie brilliant blue (DAT Ag).^2^ This test is performed by incubating the serum, diluted in a buffer containing beta-mercaptoethanol (β-ME) and albumin, with the antigen in V-bottom microtiter plates for 18 hours.^1,3^

The need for small laboratory equipment, well-trained personnel, and long incubation times makes the DAT less field applicable than the rapid diagnostic immunochromatographic test (ICT). Moreover, production of the promastigotes is subject to considerable batch-to-batch variation and is difficult to quality control.

The alternatively most widely used field-adapted point-of-care antibody detection test (short incubation, little training, and equipment needed) is an ICT based on the rK39 antigen, which is a recombinant antigen comprising 6.4 repeats of a 39–amino acid stretch of a large kinesin-related protein from an *L. infantum* isolate.^4^ Thanks to its ease of manipulation and interpretation, rapid results (15–30 minutes), and high sensitivity and specificity on the Indian subcontinent (97.0% [95% CI: 90.0–99.5%] and 90% [95% CI: 76–98%], respectively) can be obtained. It has, therefore, become the reference test for VL diagnosis in that region.^5^ Unfortunately, the sensitivity of the rK39-based ICT has been reported to be variable in East African populations (85.3%; 95% CI: 74.5–93.2%). This lack of sensitivity of the rk39 ICT has been attributed to the diversity in the sequences of the K39 gene of the *L. donovani* strains isolated in East Africa.^6,7^ Moreover, the antibody titers measured by ELISA using homologous *L. donovani* lysates have been shown to be much lower in VL patients from East Africa than in those from the Indian subcontinent.^8^ It is currently unclear why the sensitivity of the DAT is not affected by parasite strain diversity or patients’ antibody titers.

Determining which antigen(s) on the surface of the promastigotes react(s) with antibodies in patients’ blood, thus causing the agglutination reaction, would enable 1) the understanding of the mechanism whereby the DAT overcomes parasite diversity and low antibody titers in East Africa, 2) implementation of a quality control system for the production of the DAT, 3) replacement of the DAT Ag by defined synthetic or recombinant antigen(s).

We recently conducted a literature review on the existing evidence on the nature of the DAT Ag.^9^ We found that most of the evidence reported was inconclusive, as most studies were focused on improving the sensitivity and specificity of the antigen rather than determining the identity of the reacting antigen(s)/epitopes. However, based on the observations of these studies, we formulated hypotheses on the nature of the DAT Ag. In this current study, we collected experimental evidence for our two main hypotheses on the complex nature of the DAT Ag being that 1) lipophosphoglycan (LPG) and 2) carbohydrates (apart from LPG) were the main antigens recognized by VL sera in DAT.

The outer membrane of a *Leishmania* promastigote is estimated to contain 1–5 × 10^6^ molecules of LPG, covering at least 25% of the total cell surface.^10^ Lipophosphoglycan has a glycophosphatidylinositol (GPI) anchor composed of a 1-O-alkyl-2-lysophosphatidylinositol lipid anchor. It also has a heptasaccharide core (the GPI anchor and this glycan core are referred to as core–anchor fragment) linked to a long phosphoglycan (PG) polymer, composed of 15–30 repeats of Galβ 1,4 Manα 1-PO_4_ and terminated by a capping oligosaccharide (Supplemental Figure 1).^11^ Some observations suggest that LPG plays a role in the agglutination reaction in the DAT. Hommel and others^12^ showed that a monoclonal antibody against LPG reacts in the DAT. Lipophosphoglycan has a high diagnostic accuracy for detecting VL-specific antibodies in ELISA.^13^ That LPG is an antigen in the DAT reagent seems to be contradicted by the observation that LPG has a masking effect on the antibody binding to live promastigotes. Indeed, live wild-type promastigotes agglutinate less with VL patients’ serum than the promastigotes of an LPG-minus mutant; they also do not stain with VL patients’ serum in an immunofluorescent assay, whereas the LPG-minus promastigotes do.^14^ The fixation process during the DAT preparation seems to disrupt this masking effect of LPG.^14^ It is noteworthy that the LPG-minus mutant R2D2 used in these assays still harbors the core–anchor fragment of LPG.^15^ The reaction of LPG with VL-specific antibodies in the ELISA^13^ can be completely inhibited by its core–anchor fragment.^14^ These observations suggest that the core–anchor fragment of LPG is part of the DAT Ag. We hypothesized that it becomes exposed during the fixation of the promastigotes during the production of DAT, either by cleaving off the PG repeats or by a reshuffling process.^9^

Apart from LPG, the glycocalyx covering the promastigote cells contains numerous other carbohydrates: glycoproteins such as gp63 and glycoinositolphospholipids.^18^ Interestingly, β-ME treatment of promastigotes increases concanavalin A (ConA)–binding sites and also increases the sensitivity of the DAT.^19^ Based on these observations, we hypothesized that carbohydrates are likely to be part of the DAT Ag.

Verifying these two hypotheses would allow us to gather more information on the nature of the DAT Ag and may reveal the identity of the antigen. This will help further optimize the DAT and could potentially transform it into a more field-adapted ICT with broad activity across endemic regions.

## MATERIALS AND METHODS

### Ethics statements.

Sera from 16 VL patients and five healthy endemic controls were collected in previous studies: 1) “Syndromic Approach to Neglected Infectious Diseases” (NIDIAG, ethics committee [EC] FP7 contract 260260) in Gedarif, eastern Sudan, 2) “New Concepts of Simplified Diagnosis of Visceral Leishmaniasis from Peripheral Blood Samples” (VL-blood, ITG nr. 11 12 4 766) in Gondar, Ethiopia. All individuals gave their written informed consent before providing blood. Permission for the NIDIAG study was obtained from the EC of the University of Khartoum in Sudan, EC of the LSTHM (application number 5867), and EC of the University Hospital Antwerp (UZA) (Belgium registration number B300201214571). Permission for the VL blood study was obtained from the Institutional Review Board of ITMA (reference number 11124766), the UZA (Belgium registration number B300201111279), and EC in Gondar, Ethiopia (reference number RCS/05/89/2011). All specimens were anonymized.

### Parasite culture.

*Leishmania donovani* MHOM/--/SD/1-S (LD 1-S)^20^ logarithmic phase promastigotes were cultured at 26°C in GLSH (glucose, lactalbumin, serum, and hemoglobin) medium^21,22^ with 10% fetal calf serum.

### Solvent extraction of LPG from LD 1-S promastigotes.

Lipophosphoglycan was extracted as described by Orlandi and Turco^10^ (Supplemental Figure 2). The 10^9^ exponentially grown promastigotes were harvested by centrifugation and washed three times in 10 mL phosphate-buffered saline (PBS). The pellet was frozen at −20°C. Cells were extracted by addition of 2.5 mL solvent A (chloroform/methanol, 2:1) and 0.5 mL 4 mM magnesium chloride followed by sonication (five times, 60 watt, every 3 seconds for 1 minute interspaced by a 20-second break) and centrifugation. The resulting solid interphase was recovered, and the liquid phases were discarded. The solid interphase was extracted twice by addition of 2.5 mL 4 mM magnesium chloride followed by sonication and centrifugation as described earlier, after which the pellet was recovered and the supernatant was discarded. Subsequently, the pellet was extracted with 0.5 mL solvent B (chloroform/methanol, 1:1) followed by sonication and centrifugation as described earlier, twice with 1 mL solvent C (chloroform/methanol/water, 10:10:3) and three times with 1 mL solvent D (water/ethanol/diethyl ether/pyridine/concentrated ammonium hydroxide, 15:15:5:1:0.017).

### Thin layer chromatography (TLC).

Thin layer chromatography to separate the different fractions from the LPG extraction described previously was performed as described by Goossens et al.^23^ In brief, the fractions extracted with solvent B, C, and D were dried by vacuum centrifugation, and the dry weight was measured and solubilized to 10 mg/mL in chloroform/methanol (1:4). Twenty microliters of the sample was spotted on silica gel 60 TLC plates (Merck, Darmstadt, Germany) and separated in a TLC chamber using isopropanol/pyridine/water (1:1:1) as the solvent system. Glycolipids and triglycerides were detected by spraying orcinol (0.1% orcinol in 5% H_2_SO_4_) solution on the plates, drying with a hair dryer, and then charring at 110°C for 5 minutes. Triglycerides are expected to be stained in brown and hexoses in purple.

### Detection of LPG by ELISA with the monoclonal antibody CA7AE.

We used an anti-LPG mouse monoclonal IgM—CA7AE (Bio-Rad Laboratories Inc., Hercules, CA). This antibody is directed against the PG repeat epitope of LPG. The phosphor molecules have been shown to be an essential part of the epitope.^24^

Immunoplates (MaxiSorp, Nunc, Roskilde, Denmark) were coated at 4°C for 16 hours with DAT Ag (reconstituted according to the manufacturer’s description, AMC, the Netherlands, 1:40 dilution in PBS, 100 µL/well) or with the extraction fractions and solvents used for extraction (prepared as described earlier, diluted 1:10 in PBS 100 µL/well) or left empty for the no antigen (Ag0) control. Wells were blocked for 1 hour at ambient temperature (rt) with 350 µL/well PBS-blotto (1% skimmed milk). Lipophosphoglycan was detected by CA7AE monoclonal mouse IgM (Biorad,^24^ diluted 1:100 in PBS-blotto, 100 µL/well) with secondary antibody horseradish peroxidase (HRP)–conjugated AffiniPure goat anti-mouse IgM(µ) (Kirkegaard & Perry Laboratories (KPL), SeraCare, Gaithersburg, MD, 1:1,000 in PBS-0.1% Tween [PBS-T], 100 µL/well). The plates were washed three to five times with 350 µL/well PBS-T between the reactions. Color reactions were developed with 3,3′,5,5′-tetramethylbenzidin (Thermo Scientific Pierce, Waltham, MA), stopped with sulfuric acid (2 M), and measured at 450 nm. The corrected optical density (ODc) was calculated by subtracting the OD of the Ag0 control or the solvent control.

### Effects of sodium metaperiodate treatment of antigens on lectin and antibody binding.

The effects of oxidation of carbohydrates on DAT Ag and LPG were tested using a sodium metaperiodate–based assay described by Choi and coworkers for other antigens.^25^ Immunoplates were coated with DAT Ag as described earlier or LPG (fraction one extracted with solvent D, diluted 1:20 in PBS 100 µL/well) or left empty for Ag0 control and blocked as described previously. The coated antigen was oxidized using 320 µL/well of oxidation buffer (50 mM sodium acetate, 50 mM sodium metaperiodate, pH 4.7) for 1 hour at rt. Control wells were incubated with Tris-buffered saline containing 1% TBS-T instead of oxidation buffer. Effects of oxidation on ConA binding sites were assessed by HRP-conjugated ConA (Sigma-Aldrich, St. Louis, MO) (50 µg/mL, 100 µL/well in ConA buffer [10 mM Tris-HCl, pH 7.4, 0.5 M NaCl, 1 mM CaCl_2_, and 1 mM MgCl_2_]). Effects on the PG repeat epitope of LPG were assessed with monoclonal CA7AE diluted 1:100 in PBS-blotto and revealed as described earlier. Effects on epitopes reacting with specific human antibodies were detected with DAT-positive sera diluted 1:250 in PBS-blotto. Binding of human antibodies to the antigens was revealed by HRP-conjugated AffiniPure rabbit antihuman IgG (H+L) (Jackson ImmunoResearch, West Grove, PA, 1:40,000 in PBS-T). The washing, enzymatic reaction, plate reading, and calculation of the ODc were performed as described earlier.

### Inhibition Elisa

#### Antigen inhibition ELISA by preincubation of serum with the DAT antigen.

**Figure 1.**
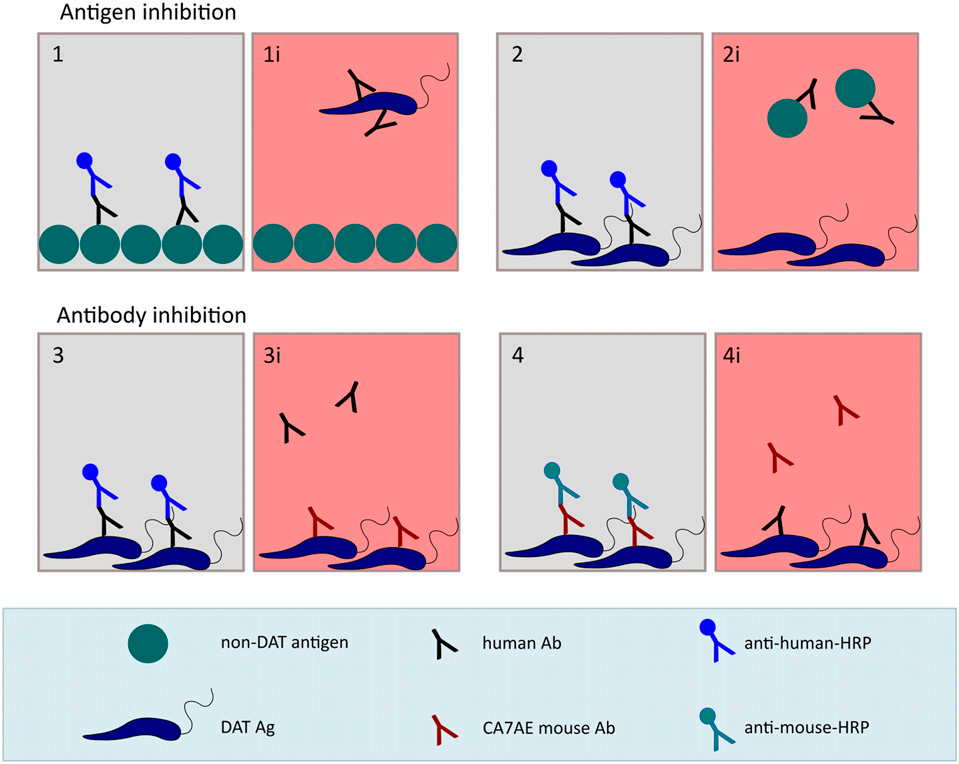
Schematic drawing of the inhibition ELISAs used in this study. Panels with red background and indicated with an i represent the inhibited version of the panel with gray background. Ab = antibody; Ag = antigen; DAT = direct agglutination test; HRP = horseradish peroxidase. This figure appears in color at www.ajtmh.org.

Lipophosphoglycan and rK39 were tested for having shared epitopes with the DAT Ag using an inhibition ELISA setup, consisting of preincubation of human sera with DAT Ag before adding them to plates coated with LPG (Figure 1, panel 1 and 1i).

Wells were coated as described earlier with LPG (1:20 dilution), rK39 (Rekom Biotech, Granada, Spain, 0.5 ng/µL, 100 µL/well), or left empty for Ag0 control and blocked as described previously. Sera were diluted 1:125 in PBS-blotto and preincubated with an equal volume of reconstituted DAT Ag. For the dose–response curve, the serum dilution was incubated with a 1:2 dilution series of DAT Ag, starting from pure reconstituted DAT Ag. Sera were preincubated with DAT Ag for 1 hour at rt and added to the plate (100 µL/well). Reactions with the conjugated secondary antibodies, washing, enzymatic reaction, plate reading, and calculation of ODc were performed as described earlier.

#### Antigen inhibition ELISA by preincubation of serum with LPG.

Lipophosphoglycans were tested for having a shared epitope with the DAT Ag by using an inhibition ELISA, consisting of preincubation of human sera with LPG before adding these to plates coated with the DAT Ag or left empty for the Ag0 control (Figure 1, panel 2 and 2i). Wells were blocked as described before. Lipophosphoglycan was titrated starting at a dilution of 1:2. The reaction with conjugated secondary antibodies, washing, enzymatic reaction, and plate reading were performed as described earlier.

#### Antibody inhibition ELISA using the CA7AE monoclonal antibody for inhibiting the reaction of serum with DAT Ag and vice versa.

To determine whether human sera react with the PG repeat epitope of LPG, human DAT-positive or -negative sera, and monoclonal antibody CA7AE were applied successively to an ELISA plate coated with DAT Ag, as described earlier (Figure 1, panel 3 and 3i, 4 and 4i). The inhibiting antibody was applied first, followed by the antibody to be detected by its specific HRP-conjugated secondary antibody. For inhibition of human sera by CA7AE, CA7AE was added in serial dilutions starting from 1:100 in PBS-blotto for 1 hour at rt 100 µL/well, followed by human serum diluted 1:250 in PBS-blotto. As a control for cross-reactivity of the antihuman HRP-conjugate with the CA7AE, a titration series of CA7AE was incubated with PBS-blotto instead of human serum. Binding of human serum to the DAT Ag was assessed by HRP-conjugated AffiniPure rabbit antihuman IgG (H+L) as described earlier. For inhibition of CA7AE by human serum, human serum was added in serial dilutions starting from 1:50 in PBS-blotto for 1 hour at rt 100 µL/well, followed by CA7AE diluted 1:400 in PBS-blotto. As a control for any cross-reactivity between the anti-mouse HRP-conjugate and human antibodies, a titration series of human serum was incubated with PBS-blotto instead of CA7AE. Binding of CA7AE to the DAT Ag was assessed with HRP-conjugated AffiniPure goat anti-mouse IgM(µ), as described previously. Reactions with conjugated secondary antibodies, washing, enzymatic reaction, and plate reading were performed as described previously. The corrected OD was calculated by subtracting the OD of the Ag0 and of the conjugate control wells.

#### Affinity purification of DAT-specific antibodies from human serum.

Direct agglutination test–specific antibodies were purified from DAT-positive and -negative sera originating from Ethiopia and Sudan as follows. The DAT titer of the serum samples was evaluated according to the kit’s instructions (DAT ITMA).^26^ Sera were considered DAT negative with a DAT titer < 1:100. Direct agglutination test–positive sera used in this study showed a DAT titer > 1:51,200. Two and a half milliliters of serum, diluted 1:100 in DAT diluent buffer (ITMA, Belgium), was incubated with 2.5 mL of DAT Ag (ITMA, Belgium, reconstituted according to the manufacturer’s instructions) on a roller at rt overnight. After five washes with 800 µL DAT buffer (ITMA, Antwerp, Belgium) (centrifugation at 2,000 × *g* for 5 minutes), antibodies bound to the DAT Ag were eluted with 700 µL of 0.2 mol/L glycine/HCl, pH 2.6, for 10 minutes and subsequently neutralized with 100 µL of 1 mol/L Tris/HCl, pH 9.1.

Direct agglutination test–specific antibodies were tested in the DAT according to the kit’s instructions (DAT ITMA), but starting with dilution 1:2.^26^ The antibodies were also quantified using ELISA. In brief, ELISA plates were coated for 16 hours at 4°C with 100 µL/well of 1:2 serial dilutions of serum/antibodies, with a starting dilution of 1:200 for serum and 1:2 for antibody fraction in PBS. Two wells were left empty as an antibody-negative control (Ab0). A serial dilution of a purified immunoglobulin (Ig) standard with a starting concentration of 9 µg/mL was added as a standard curve. Immunoglobulins of the standard were isolated using protein L chromatography cartridges (Thermo Scientific Pierce, Waltham, MA) according to the manufacturer’s instructions, and the Ig concentration was quantified using a BCA Protein Assay Kit (Pierce^™^) with a bovine gamma globulin standard following the manufacturer’s instructions. Reactions with the conjugated secondary antibodies, washing, enzymatic reaction, and plate reading were performed as described earlier. The measured OD was corrected (ODc) with the corresponding OD in the Ab0 wells.

Direct agglutination test–specific antibodies were tested for their reactivity with LPG by adding 100 µL of the isolated DAT-specific antibodies to an ELISA plate coated with LPG as described earlier. Reactions with conjugated secondary antibodies, washing, enzymatic reaction, and plate reading were performed as described previously.

### Statistical analyses.

A Welch two-sample *t*-test was performed in R.^27^

## RESULTS

### Lipophosphoglycan as part of the DAT Ag.

To verify whether LPG, more precisely its PG repeat epitope, is exposed on the DAT Ag, we tested the reactivity of the monoclonal antibody CA7AE, directed against the PG repeat epitope of LPG, using an ELISA with the DAT as antigen and in DAT format. In the ELISA, CA7AE reacted at an average ODc_450nm_ of 1.09 (±0.55) (Supplemental Figure 3A), and the end-titer of CA7AE in DAT was 1:400. The PG repeat epitope of LPG is, thus, exposed on the DAT Ag.

The same monoclonal antibody was used to test the different organic solvent extracts of *L. donovani* promastigotes for the presence of LPG. The ELISA results show that CA7AE reacted strongly with solvent D extracts (median ODc_450 nm_ 1.52) and to a lesser extent with solvent B and C extracts (median ODc_450 nm_ 0.33, 78% less than solvent D extracts) (Supplemental Figure 4B). In addition, TLC revealed a condensed spot in solvent D and a broader spot of glycolipids in solvent B and C (Supplemental Figure 4). As a single, condense spot was visible on TLC with solvent D extracts, we concluded that there were no major contaminants present in this extract. Thus, based on the ELISA and TLC results, we consider the solvent D extract as LPG from hereon.

**Figure 2.**
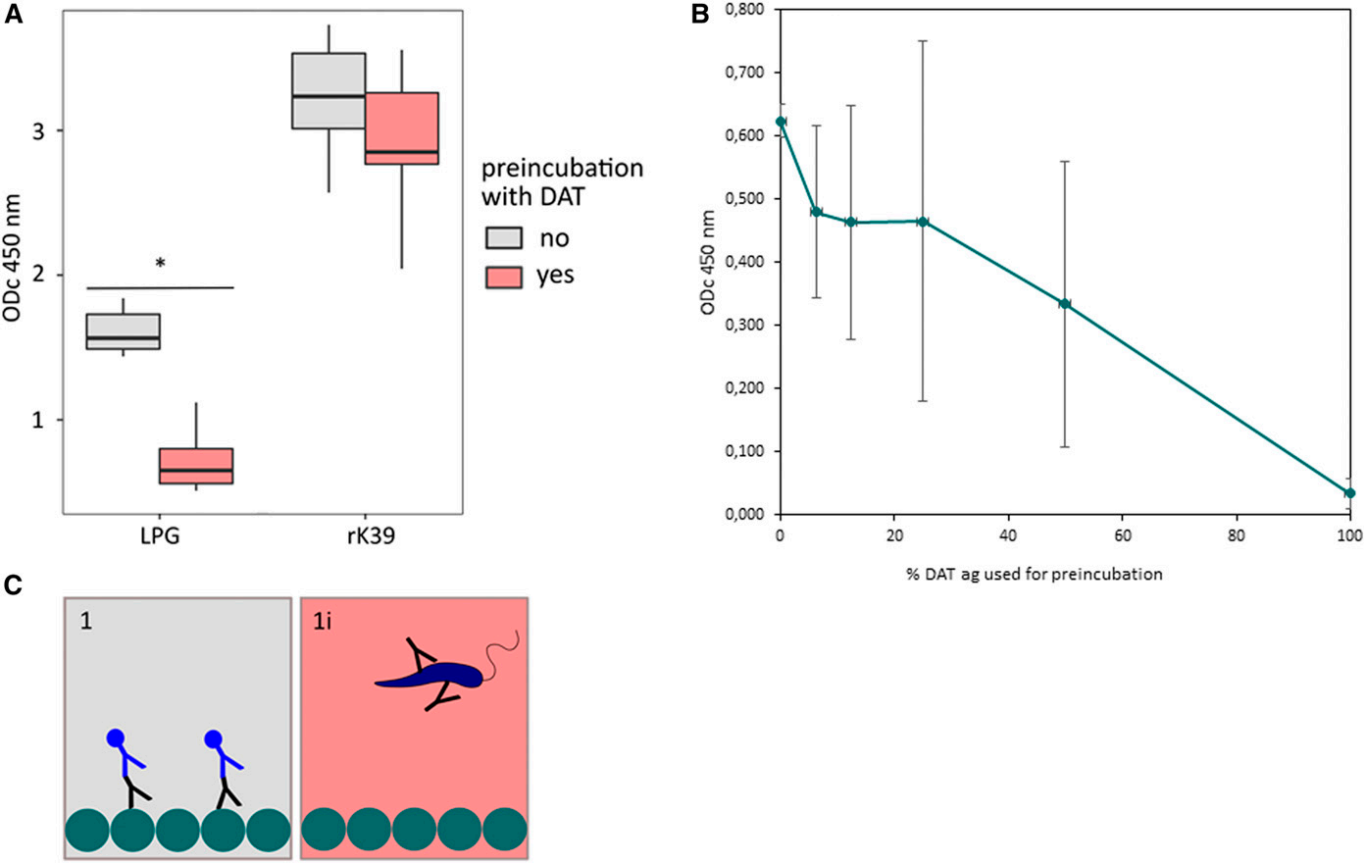
Effect of preincubation of sera with direct agglutination test (DAT) Ag on reactivity with lipophosphoglycan (LPG) and rK39 in ELISA. Results of five DAT-positive sera with three replicates per serum with (red) and without (gray) preincubation with DAT Ag **(A)**. Results of two DAT-positive sera preincubated with different dilutions of DAT Ag before ELISA with LPG as Ag, 100% = pure reconstituted DAT Ag **(B)**. Inhibition assay used as depicted in Figure 3 (**C**). *P < 0.01. This figure appears in color at www.ajtmh.org.

To assess whether LPG and DAT Ag share epitopes, we investigated whether LPG reacts with DAT-positive sera and whether this reaction can be inhibited by preincubating the sera with DAT Ag. As shown in Figure 2A, purified LPG and rK39, as control, react strongly with DAT-positive sera (*n* = 5, median ODc_450nm_ 1.56 and 3.24, respectively) in the ELISA. Preincubating the sera with DAT Ag significantly inhibits the reaction of the DAT-positive sera with LPG (median ODc_450nm_ 0.65; 80% inhibition, *P* < 0.05) but to a much lesser extent with rK39 (median ODc_450nm_ 2.85; 12% inhibition, *P* > 0.05). This inhibition effect depends on the concentration of DAT used as shown in the dose–response curve in Figure 2B.

**Figure 3.**
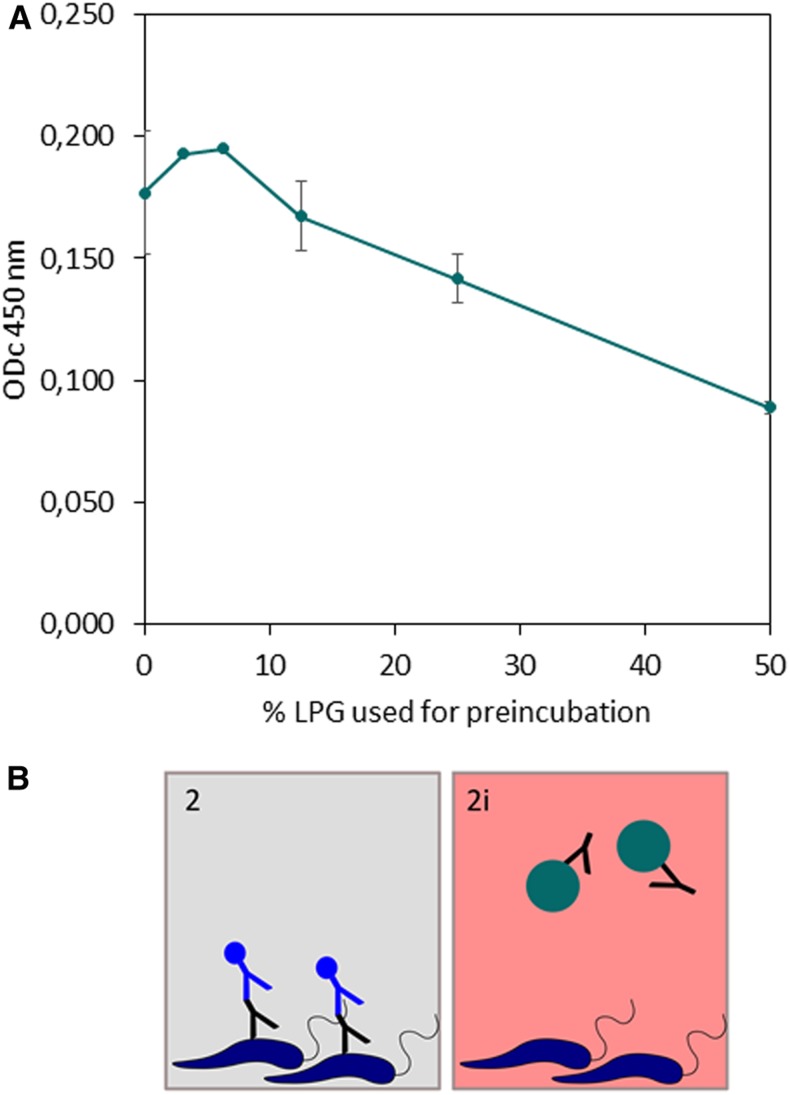
Effect of preincubation of DAT-positive (*n* = 2) sera with different dilutions of lipophosphoglycan (LPG) on reactivity with DAT Ag in ELISA. Results of three repetitions per serum, 100% = pure reconstituted LPG (**A**). Inhibition assay used as depicted in Figure 1 (**B**). This figure appears in color at www.ajtmh.org.

In a reciprocal way, preincubating sera from DAT-positive VL patients with increasing amounts of LPG before an ELISA with DAT as antigen (as depicted in Figure 1, panel 2 and 2i) partly inhibits the sera’s reactivity with DAT Ag in a dose-dependent manner (from ODc_450 nm_ 0.177 to 0.089, 49% inhibition) (Figure 3).

**Figure 4.**
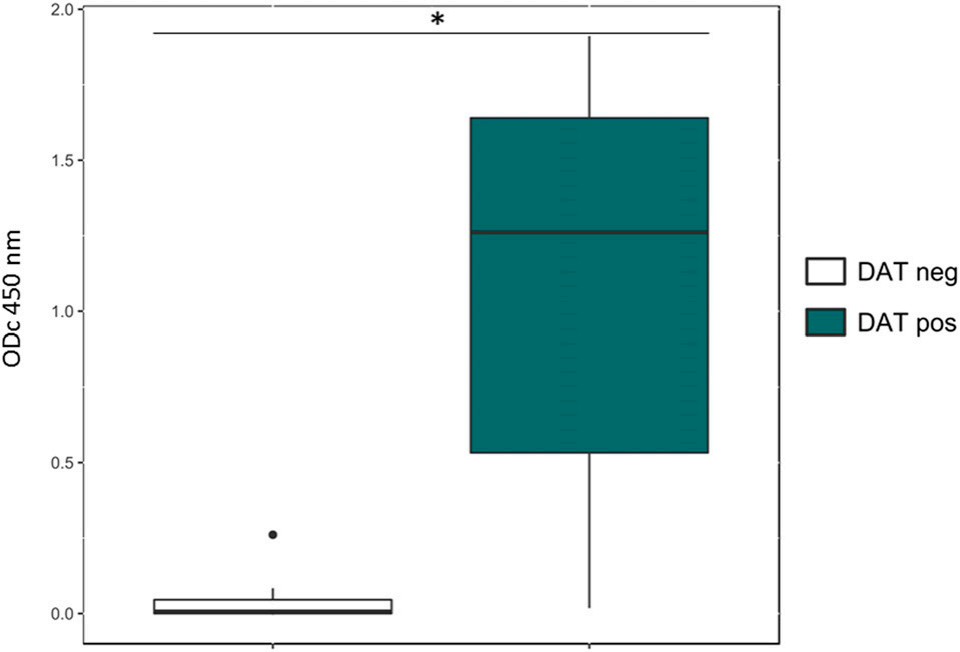
Reaction of affinity-purified DAT-specific antibodies from 16 visceral leishmaniasis patients and eight healthy controls with lipophosphoglycan in ELISA. *P < 0.05. This figure appears in color at www.ajtmh.org.

Further evidence that DAT Ag and LPG share epitopes was gained from the experiments with human serum antibodies affinity-purified on DAT Ag. Affinity-purified antibodies from DAT-positive VL patients still caused agglutination in the DAT test, whereas antibodies from healthy controls (VL−), purified following the same procedure, did not (Supplemental Figure 5). Also in the ELISA with LPG as antigen, the purified DAT-specific antibodies from VL patients were significantly more reactive (median ODc_450 nm_ 1.17, *P* < 0.05) than purified antibodies from healthy controls (median ODc_450 nm_ 0.01) (Figure 4).

From the aforementioned experiments, we conclude that DAT Ag and LPG share epitopes that react with antibodies in sera from VL patients.

**Figure 5.**
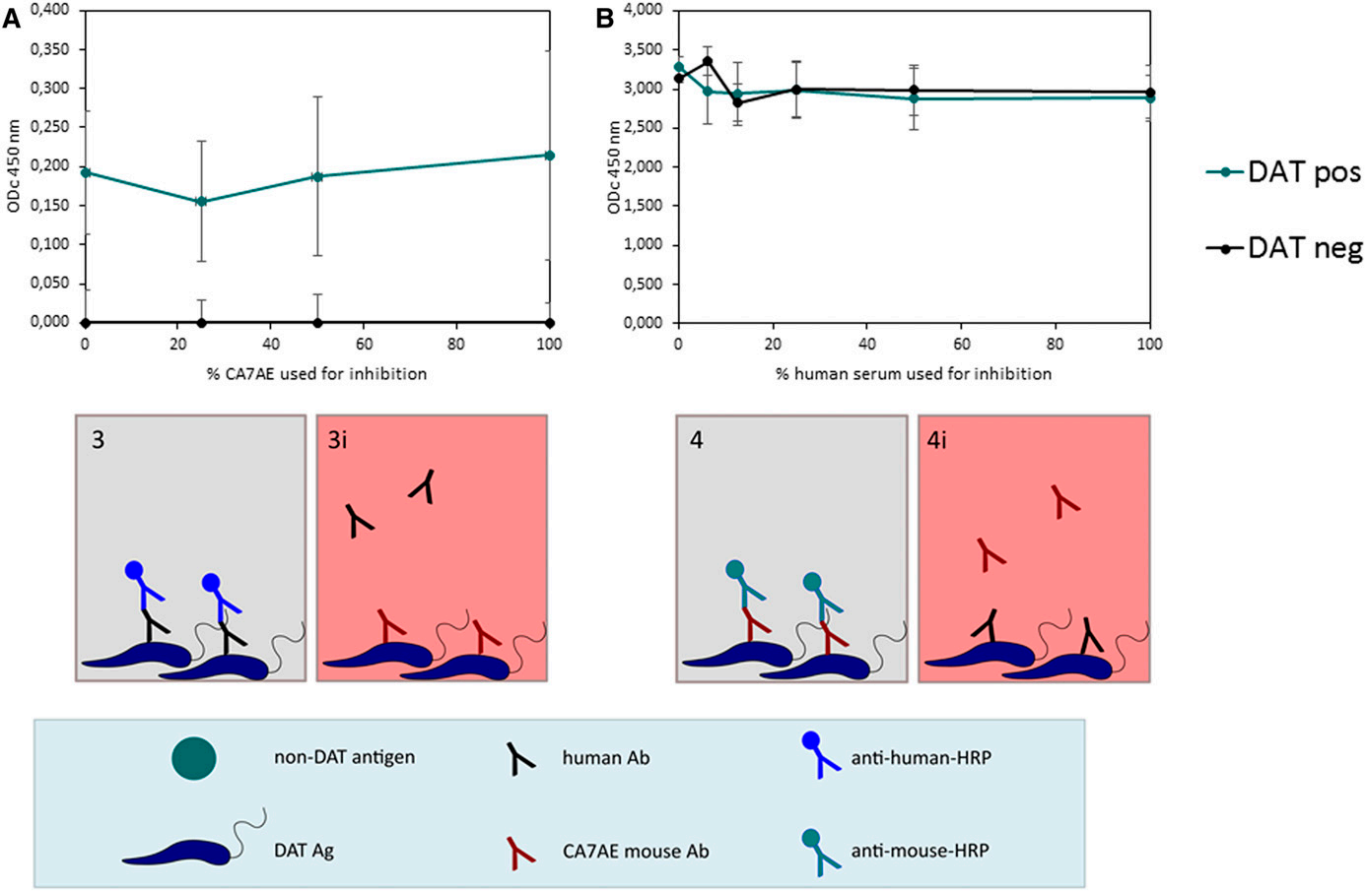
Effect of antibody inhibition on the reaction of human sera or CA7AE with DAT Ag. (**A**) Top: ELISA coated with DAT Ag reacted first with serial dilutions of CA7AE and second with human visceral leishmaniasis (VL)-positive (*n* = 4) and -negative sera (*n* = 2), 100% = 1:100 dilution of CA7AE; bottom: inhibition assay used as depicted in Figure 2. (**B**) Reciprocal assay. Top: ELISA coated with DAT Ag reacted first with serial dilutions of human VL-positive (*n* = 3) and -negative sera (*n* = 3) and second with CA7AE), 100% = 1:250 dilution of serum; bottom: inhibition assay used as depicted in Figure 1. This figure appears in color at www.ajtmh.org.

To determine whether antibodies in DAT-positive VL patient sera and the PG-specific monoclonal antibody CA7AE react with the same epitope on the DAT Ag, we conducted two competition ELISAs. Figure 5A shows that applying increasing amounts of CA7AE to DAT Ag–coated microtiter plate does not affect the reactivity of human sera with the DAT Ag in ELISA. Also, no inhibition effect on the reactivity of CA7AE is observed when, in a reciprocal way, the coated microtiter plate is preincubated with increasing amounts of DAT-positive and DAT-negative sera (Figure 5B). These data suggest that most antibodies in sera from VL patients do not react with the mouse monoclonal CA7AE epitope on DAT Ag.

**Figure 6.**
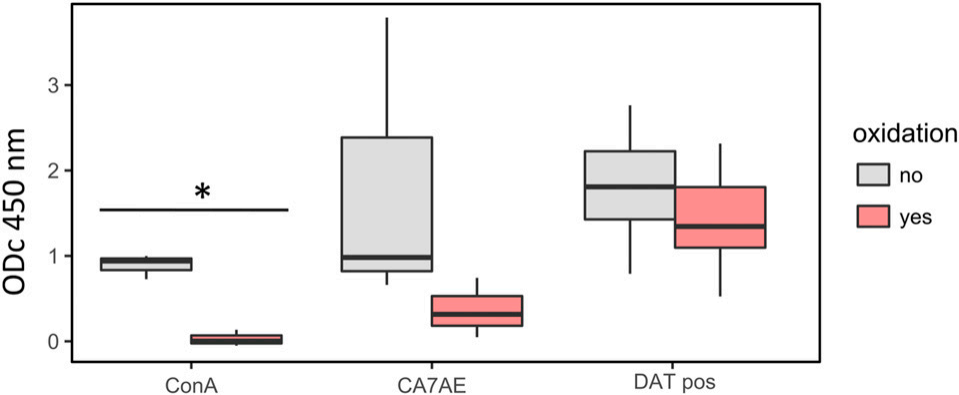
Effect of sodium metaperiodate oxidation of lipophosphoglycan on its reactivity in ELISA with concanavalin A (ConA), with the anti-PG repeat monoclonal antibody CA7AE, and with DAT-positive sera (*n* = 5) from visceral leishmaniasis patients. *P < 0.05. This figure appears in color at www.ajtmh.org.

To assess whether the carbohydrate moiety of LPG is responsible for binding antibodies of VL patients, we oxidized the carbohydrates exposed on LPG using sodium metaperiodate. As controls, we used the lectin ConA, known to bind to α-D-mannosyl and α-D-glucosyl residues, as well as CA7AE, known to bind the PG epitope of LPG. Oxidation of LPG with sodium metaperiodate almost completely abolished the binding of ConA (from median ODc_450 nm_ 0.94 to 0.03, decrease of 99%) (Figure 6). The effect on the binding of CA7AE was less pronounced (from median ODc_450 nm_ 0.99 to 0.32, decrease of 68%). The decrease in reactivity with DAT-positive human sera was not significant (from median ODc_450 nm_ 1.85 to 1.35, decrease of 27%), suggesting that carbohydrates, at least the ConA-binding sites including the CA7AE epitope and the heptasaccharide core, are not the major epitope of LPG recognized by sera from VL patients.

#### Carbohydrates as part of the DAT Ag.

**Figure 7.**
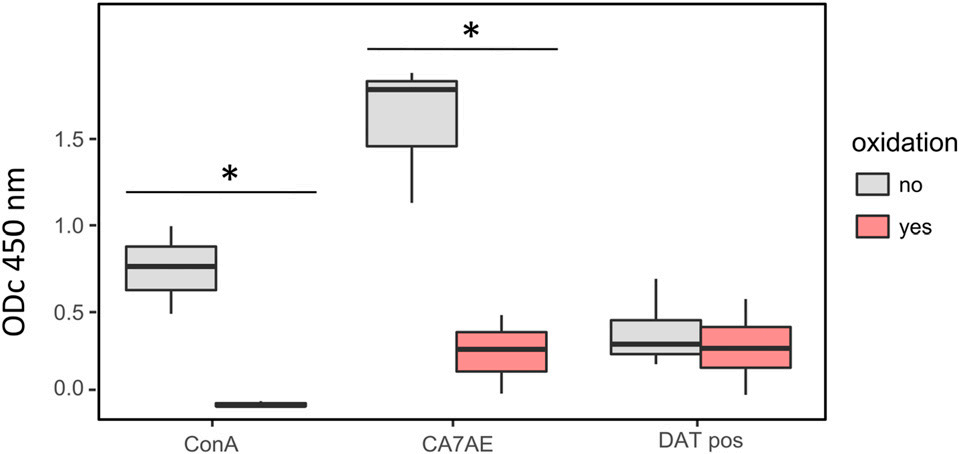
Effect of sodium metaperiodate oxidation of DAT on its reactivity in ELISA with concanavalin A (ConA), with the anti-PG repeat monoclonal antibody CA7AE, and with DAT-positive sera (*n* = 5) from visceral leishmaniasis patients. *P < 0.05. This figure appears in color at www.ajtmh.org.

To verify our second hypothesis, that is, carbohydrates (other than LPG) being part of the DAT Ag, we used sodium metaperiodate to oxidize the entire DAT Ag. Oxidation of DAT Ag coated in an ELISA plate resulted in a complete loss of reactivity with ConA (from median ODc_450 nm_ 0.77 to −0.04) and a significant decrease of reactivity with CA7AE (from median ODc_450 nm_ 1.79 to 0.9, 84% decrease), whereas the reaction with DAT-positive sera decreased only by 8% (median of all DAT-positive sera from ODc_450 nm_ 0.29 to 0.27) (Figure 7). These data suggest that most epitopes reacting with antibodies from VL- patients in DAT are different from the epitope recognized by CA7AE (PG repeats of LPG) and from the ConA-binding sites on DAT Ag. They also suggest that no or only a small part of DAT epitopes recognized by positive sera bear carbohydrate moieties.

## DISCUSSION

Karp et al.^14^ described a shielding effect of LPG against antibody binding to live promastigotes, which is reversed during the production process of DAT. Previously, we hypothesized that the PG repeat epitope of LPG is cleaved off during formaldehyde treatment of the *L. donovani* promastigotes, thus abolishing this shielding effect (Supplemental Figure 6A).^9^ Here, we show a reaction of the monoclonal antibody CA7AE with the DAT Ag, both in the DAT and in the ELISA. This demonstrates that the PG epitope is still present on the DAT Ag, suggesting that the LPG coat on the promastigote cells is rendered porous to antibodies by a reshuffling process (Supplemental Figure 6B). However, the fact that PG epitopes are exposed on the surface of the DAT Ag does not suffice to conclude that LPG is involved in the DAT reaction with serum of VL patients. The evidence that DAT Ag and LPG share diagnostic epitopes comes from our results showing that the reaction of VL patients’ sera with purified native LPG in ELISA can be inhibited by preincubation of the serum with DAT Ag. On the other hand, the inhibition experiments with DAT Ag coated in ELISA microtiter plates revealed that preincubation with neither the monoclonal CA7AE nor with VL patient sera leads to a decreased reactivity of, respectively, VL patient sera or CA7AE, suggesting that human sera and CA7AE do not react with the same epitope of LPG. Moreover, oxidation of the carbohydrate moiety of LPG coated in ELISA microtiter plates decreased the reactivity of LPG with CA7AE but not with VL patient sera, providing further evidence that neither the PG epitope nor other carbohydrates of LPG are involved in the DAT reaction. Because all the cap and the remaining part of the PG repeat epitope of LPG are composed of carbohydrates (Supplemental Figure 1), this leaves the phosphate and the 1-O-alkyl-2-lysophosphatidylinositol lipid anchor of the core–anchor fragment of LPG as a possible epitope involved in DAT agglutination. This corroborates the observation of Karp and others^14^ that the reactivity of human sera with LPG in ELISA (which differs between VL-positive and -negative patients)^13^ could completely be inhibited by its core–anchor fragment. While seemingly being buried under the cap and PG repeats of LPG, the core–anchor fragment has been associated with immunity. In immunization studies, LPG from *Leishmania major* showed protection from cutaneous leishmaniasis in mice. This protection was dependent on the core–anchor fragment, as the water-soluble form of LPG lacking the core–anchor fragment was not protective and even exacerbated disease.^25,26^

By preincubation of VL patients’ sera with DAT Ag, we can almost completely inhibit the reaction of serum with LPG. When we preincubate these sera with LPG, it only slightly affects their reaction with DAT Ag. This suggests that there is a mixture of epitopes reacting with sera from VL patients and could also explain why the sensitivity of DAT is less affected by *L. donovani* strain diversity in different geographic regions than the single antigen tests based on rK39. Our additional experiments demonstrated that preincubation with DAT Ag does not inhibit the reaction of sera with rK39 in the ELISA. The observation that DAT and rK39 do not share epitopes could facilitate assay development, where the antigen consists of a combination of rK39 and a DAT-specific Ag.

Results obtained in the ELISA after oxidation of the LPG and DAT Ag with sodium metaperiodate show that their reactivity with ConA and CA7AE was drastically decreased, although their reactivity with VL patient sera remained largely unaffected. These results suggest that the main diagnostic epitopes on the DAT and LPG are not of carbohydrate nature.

One might argue that testing inhibition and oxidation directly in DAT format would be more conclusive. Unfortunately, this was not feasible for multiple reasons: 1) The composition of solvent D containing LPG is incompatible with the specific agglutination reaction in DAT, 2) both CA7AE and ConA agglutinate DAT Ag without the presence of antibodies, making it impossible to assess antibody and lectin inhibition in DAT format, and 3) sodium metaperiodate oxidation of DAT Ag in suspension caused uncontrollable losses of Ag during washing steps. We did not use serum samples from the Indian subcontinent because we did not have access to a serum bank from that region. Instead, we used specimens from East Africa, because our aim was to support the development of alternative antigens for VL detection where the sensitivity of rK39-based RDTs is variable.

## CONCLUSION

In this study, we provide strong evidence that the noncarbohydrate moieties of LPG play a role in the agglutination reaction of VL patient sera with the DAT Ag. Based on the known structure of LPG, we conclude that the core–anchor fragment of LPG would be a good candidate antigen for developing a new antibody detection test for VL, in the form of the synthetic LPG core–anchor described by Ruda et al.^28^ or other non-native equivalents. We believe the way forward to improve diagnosis and patient care in East Africa is to replace the DAT by such a non-native (i.e., easily standardized) antigen incorporated in an ICT format point-of-care test.

## Supplemental figures

Supplemental materials
