# Supplementary material for: Experimental Evidence on the Nature of the Antigen in the Direct Agglutination Test for Visceral Leishmaniasis
Source: Am J Trop Med Hyg. 2020 Mar 2;102(4):788–96. doi: 10.4269/ajtmh.19-0784 (PMC7124922; doi:10.4269/ajtmh.19-0784)
Supplement: Supplementary file 1 [file tpmd190784.SD1.pdf]

Supplemental Figures 1-6

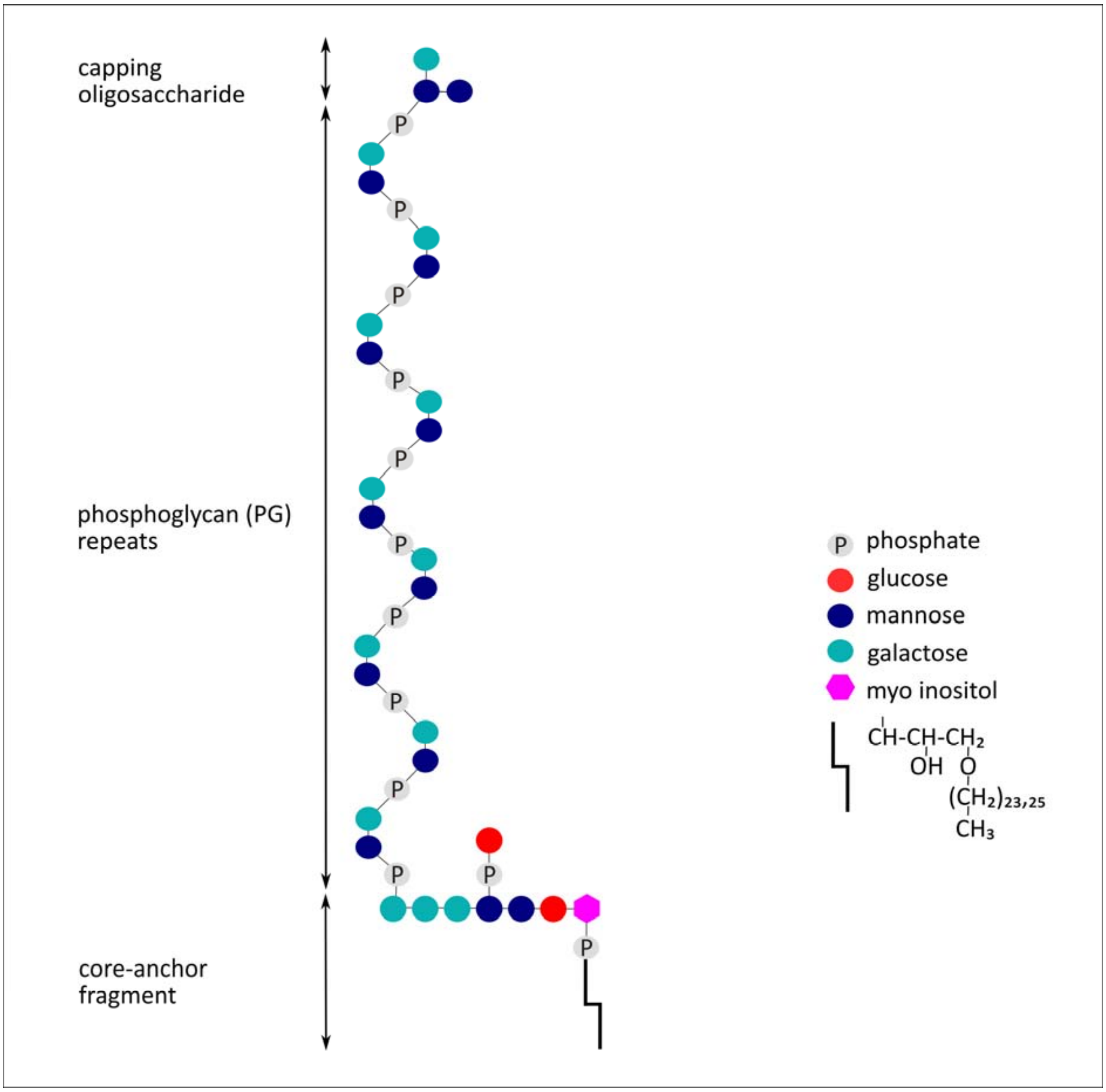

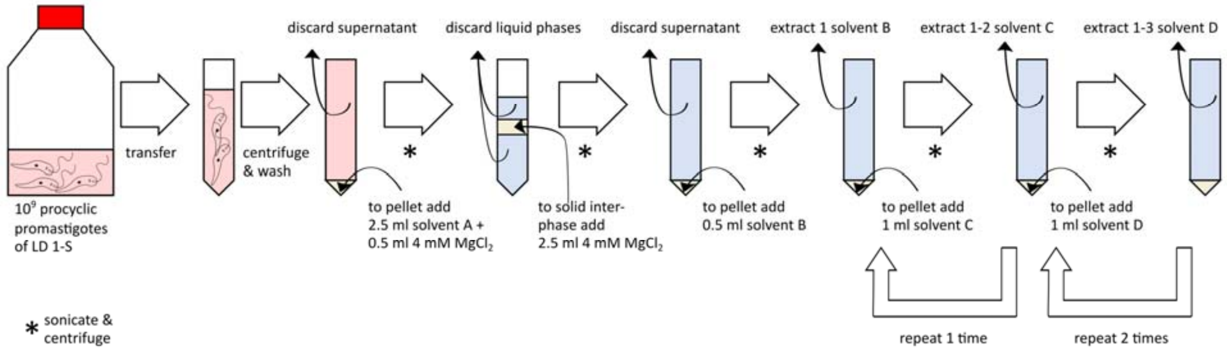
discard supernatant
discard liquid phases
discard supernatant
extract 1 solvent B
extract 1-2 solvent C
extract 1-3 solvent D
transfer
centrifuge & wash
10[9] procyclic promastigotes of LD 1-S
to pellet add 2.5 ml solvent A + 0.5 ml 4 mM MgCl2
to solid inter-phase add 2.5 ml 4 mM MgCl2
to pellet add 0.5 ml solvent B
to pellet add 1 ml solvent C
to pellet add 1 ml solvent D
repeat 1 time
repeat 2 times
* sonicate & centrifuge

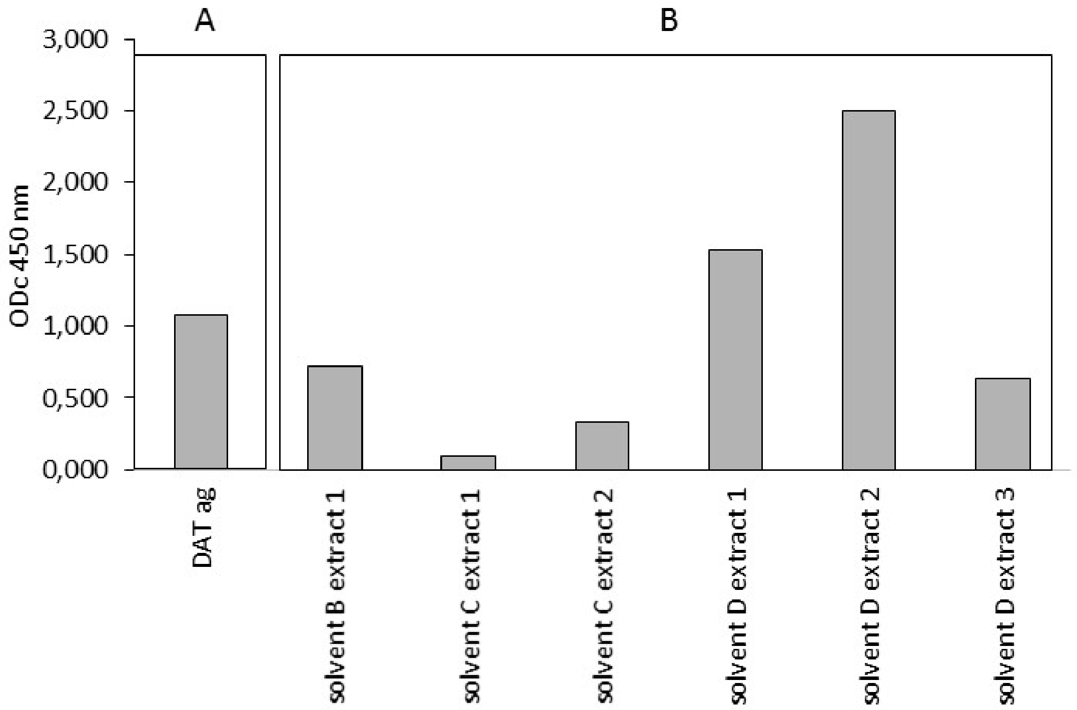
A
B
3,000
2,500
2,000
1,500
1,000
0,500
0,000
ODc 450 nm
DAT ag
solvent B extract 1
solvent C extract 1
solvent C extract 2
solvent D extract 1
solvent D extract 2
solvent D extract 3

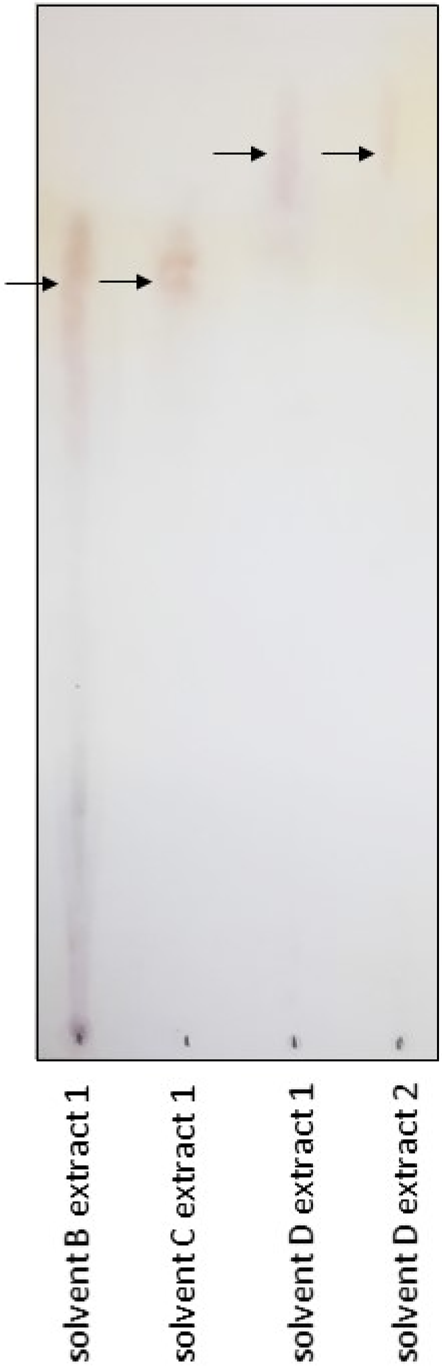
solvent B extract 1
solvent C extract 1
solvent D extract 1
solvent D extract 2

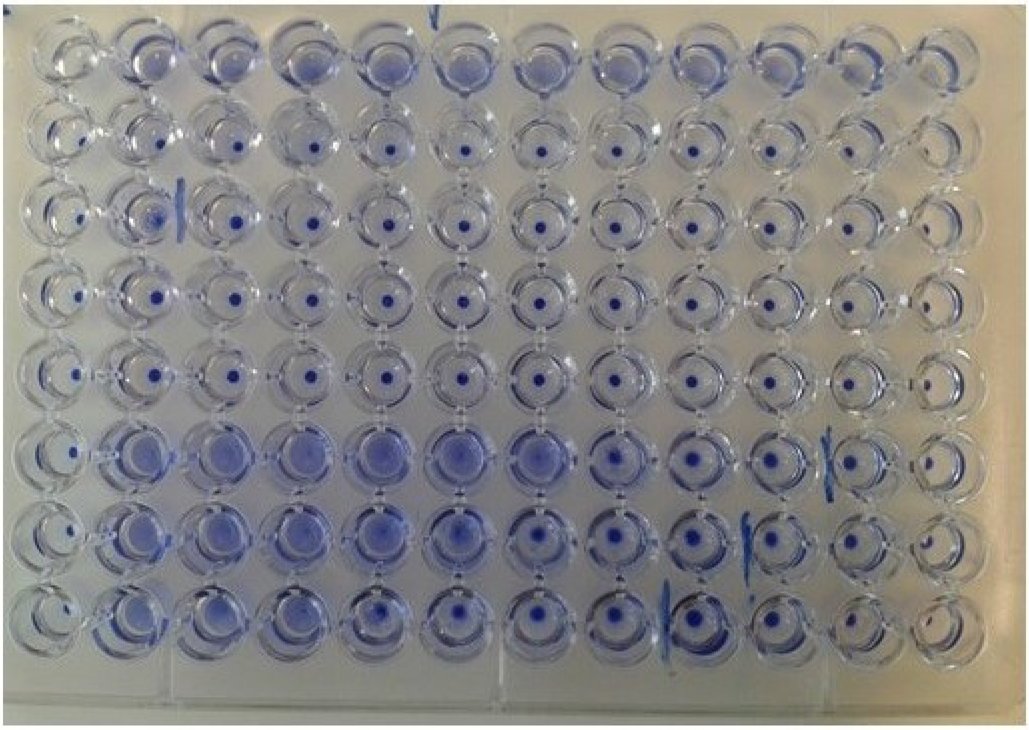

A
B
C
D
E
F
G
H

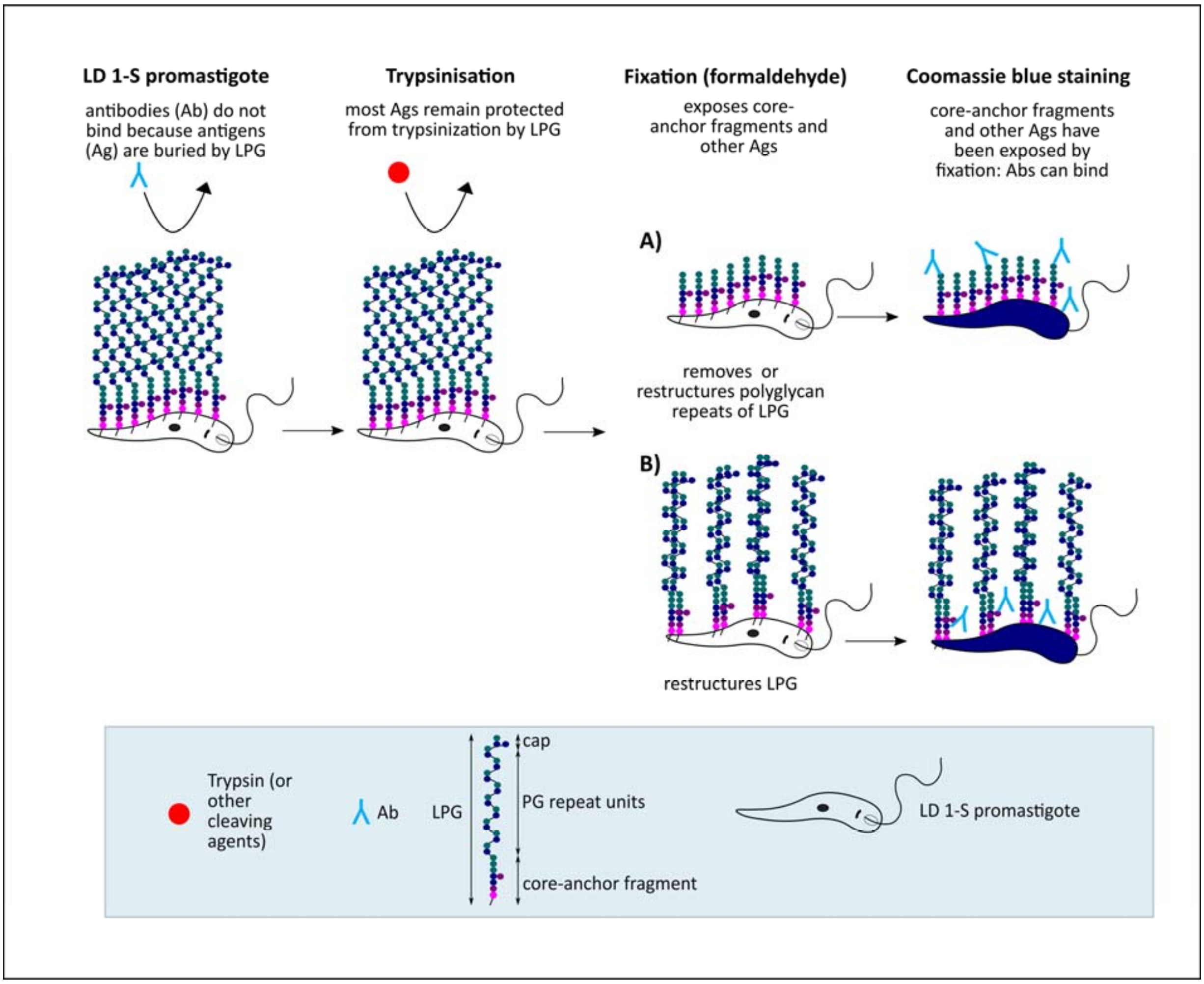
LD 1-S promastigote
antibodies (Ab) do not bind because antigens (Ag) are buried by LPG
Trypsinisation
most Ags remain protected from trypsinization by LPG
Fixation (formaldehyde)
exposes core-anchor fragments and other Ags
Coomassie blue staining
core-anchor fragments and other Ags have been exposed by fixation: Abs can bind
A)
removes or restructures polyglycan repeats of LPG
B)
restructures LPG
Trypsin (or other cleaving agents)
Ab
LPG
cap
PG repeat units
core-anchor fragment
LD 1-S promastigote

Supplemental data figure S1: Schematic drawing of LPG from a procyclic promastigote of *Leishmania donovani* from Sudan (1-S strain) as used for DAT production. [15,16]

Supplemental data figure S2: Flow chart of solvent extraction procedure

Supplemental data figure S3: Reactivity in ELISA of monoclonal antibody CA7AE with DAT Ag (A) and fractions from LD-1S extracted with solvents C and D (B).

Supplemental data figure S4: TLC separated fractions from LD-1S extracted with solvent C and D stained with orcinol in order to detect glycolipids and triglycerides.

Supplemental data figure S5: Agglutination reaction of DAT-affinity purified antibodies from 3 negative control sera (rows C-E) and from 3 VL patient sera (rows F-H). Rows A and B contain respectively the positive and negative control of the DAT kit.

Supplemental data figure S6: Schematic drawing comparing two hypotheses on LPG changes during the preparation of the DAT Ag. Fixation with formaldehyde A: completely removes the PG repeats of LPG, 8 or B: restructures the PG repeats of LPG. Ab = antibody; Ag = antigen; LD = *Leishmania donovani*; LPG = lipophosphoglycan; PG = polyglycan.
